# The Future Looks Good: Examining the Associations Between Coping, Psychological Distress, and Optimism

**DOI:** 10.3389/fpsyg.2022.838835

**Published:** 2022-05-02

**Authors:** Tiago Santos, António de Sousa Uva, José Fernandes Rodrigues, Regina Ferreira, Diogo Monteiro, Antonio Hernández-Mendo, Filipe Rodrigues

**Affiliations:** ^1^Sport Sciences School of Rio Maior, Polytechnic Institute of Santarém, Rio Maior, Portugal; ^2^National School of Public Health, New University of Lisbon, Lisbon, Portugal; ^3^Quality of Life Research Center (CIEQV), Santarém, Portugal; ^4^Health Science School, Polytechnic Institute of Santarém, Santarém, Portugal; ^5^ESECS Polytechnic Institute of Leiria, Leiria, Portugal; ^6^Research Centre in Sports Sciences, Health Sciences and Human Development (CIDESD), Vila Real, Portugal; ^7^Department of Social Psychology, Social Work, Anthropology and East Asian Studies, University of Málaga, Málaga, Spain

**Keywords:** adaptive, maladaptive, depression, anxiety, stress, optimism

## Abstract

The recent pandemic and consequent lockdown had a substantial impact on mental health and optimism regarding the future. Previous research showed that levels of depression, anxiety, and stress had increased throughout the pandemic. Nonetheless, how individuals cope when faced with adversity may be associated with positive expectations regarding the future. A sample of 274 Portuguese workers (female = 54) with a mean age of 40.86 (SD = 0.70) and work experience of 19.68 years (SD = 12.07) met inclusion criteria and agreed to participate in the proposed study. They represented a variety of working classes (i.e., arts, engineering, marketers, transportation and logistic, clerks, personal trainers, managers, lawyers, accountants, directors, journalism, health care). We investigated the associations between depression, stress, anxiety, adaptive and maladaptive coping, and optimism while controlling for working experience, gender, and work type. We found that depression was related to lower levels of optimism. However, for participants scoring high on adaptive coping and anxiety, higher scores of optimism were reported. Gender, work type, and experience did not significantly influence the results. These results provide evidence through which positive mental health can be promoted after the pandemic.

## Introduction

The psychological issues accompanied by the COVID-19 pandemic are emerging as serious public health concerns affecting individuals worldwide (Duan and Zhu, 2020). Prior reports have shown that depression, anxiety, and stress have increased throughout the pandemic. Research showed that during outbreaks and lockdown, healthcare and essential workers presented higher rates of psychological distress compared to the general population (Naser et al., 2020; Bell et al., 2021).

The psychological distress may be associated with errors and lapses in professionalism as well as a high risk of serious mental health issues such as severe depression, burnout, or suicidal intentions (Milner et al., 2018; Jun et al., 2019). Thus, it is important to shed the light on the coping strategies and the factors, which could impact workers to ensure the mental well-being they need. The importance of recognizing optimism, through perseverance and effort to obtain desired goals cannot be understated as this condition can result in a substantial increase in perceived quality of life.

### Theoretical Background

Psychological distress is a general term used to describe indicators that impact an individual level of functioning. Depression and anxiety, as well as mixed anxiety-depression dimension, are manifestations of psychological distress (Clark and Watson, 1991). While previous studies highlighted the evidence that anxiety and depression are difficult to differentiate empirically, some research analyzed measures of anxiety and depression, as well as non-discriminating anxiety and depression items, referring to difficulty relaxing, nervous tension, irritability, and agitation, labeled as stress (Lovibond and Lovibond, 1995). Lovibond and Lovibond (1995) characterize depression as the loss of self-esteem and motivation, associated with the perception of low probability of achieving goals of life that are meaningful to the individual as a person. Anxiety highlights the links between persistent anxiety states and responses and intense fear of not corresponding to expectations. Stress suggests persistent states of excitement and tension, with a low resistance to frustration and delusion (Lovibond and Lovibond, 1996).

Past research shows that psychological distress experienced in the work context may be negatively related to well-being and quality of life and these findings have been observed in a variety of work activities during and after the COVID-19 pandemic, such as health caregivers (Lenzo et al., 2021), undergraduate students (Mishra et al., 2021), and teaching and administration personnel (Salazar et al., 2021).

In addition, the strategies people use to adapt to adverse circumstances and events have the potential to impact, positively or negatively, the mental health of people, with the ability to modify the evolution of psychological distress, either avoiding the stressful situation or working toward well-being on the long-term (Lazarus and Folkman, 1984). Hence, there is evidence that depression, anxiety, and stress are significantly associated with coping, depending on the cope mechanisms adopted by the individual (Carver and Smith, 2010).

Carver and Smith (2010) define coping as efforts expended to avoid or to lessen threats, damage, loss, or to reduce associated stress. The authors claim that the identification of the nature of the responses is not easy since they can start strategically and intentionally, but with repetition, they can become automatic. Thus, an individual who must deal with adversity (e.g., COVID-19 pandemic) is engaged in some sort of coping (Carver and Smith, 2010). Depending on several factors, such as context and personality, there is sufficient empirical evidence that points out which coping strategies are the most commonly related to psychological distress or well-being (Su et al., 2015). Several authors (Meyer et al., 2001; Teques et al., 2018) classified coping strategies as maladaptive coping and adaptative coping. According to the mentioned authors, maladaptive coping includes substance use, venting, behavioral disengagement, denial, self-distraction, and self-blame. On the other hand, adaptive coping comprises positive reframing, active coping, acceptance, religion, planning and seeking social support, use of emotional and instrumental support, and humor.

There is evidence pointing toward significant correlations between coping mechanisms and mental health and well-being indicators. Meyer et al. (2001), found adaptative coping strategies to be positively related to psychological well-being in patients with severe mental illness (Meyer et al., 2001). Almeida et al. (2021) concluded that adaptive coping was positively correlated with satisfaction with life, while negatively associated with depressive symptoms (Almeida et al., 2021). On the other hand, maladaptive coping was positively correlated with depressive symptoms, and negatively associated with satisfaction with life. Likewise, García et al. (2018) found active coping and acceptance to be positively related to well-being, and negatively related to stress (García et al., 2018). Kim and Seidlitz (2002) found denial coping (part of maladaptive coping or dysfunctional coping) to be a positive determinant of stress and negative affect (Kim and Seidlitz, 2002). Bearing this in mind, coping has evolved throughout human development and has an important role in psychological and well-being adjustment. In this regard, it is understood that cope can be a significant tool for helping individuals to deal with adverse events and cope against psychological distress, and create a mental attitude characterized by faith and confidence regarding a positive future.

Optimism is the tendency to expect positive outcomes in uncertain situations (Scheier and Carver, 1985). These authors began to study optimism systematically based on scientific studies and advocated a conceptualization of optimism based on the positive expectations that the individual has about their future, while pessimism would be associated with expectations people’s negative attitudes toward their future or a situation that may occur in the future. Optimism is seen as a cognitive characteristic (expectation, belief, or a causal attribution) regarding the desired future and sense of success (Peterson, 2000). Hence, it is expected that individuals who use adaptive coping strategies related to the pandemic would reflect positive expectations that things good things will happen in the future. On the other hand, individuals who are assuming more maladaptive coping strategies would be more prone to display lower scores of optimism. Additionally, higher scores of psychological distress would present lower scores of optimism, since an increase in feelings of nervousness, anxiety, and depression, may be a consequence of dealing with decreased well-being (Smida et al., 2021).

### Current Research

While there is some existing literature regarding the associations between psychological distress, cope, and optimism (Ferreira et al., 2021; Jarego et al., 2021; Smida et al., 2021), far less information is available, however, concerning the hypothesis that coping serves as the vehicle by which psychological distress indicators are diminished leading to greater optimist experience, especially after the pandemic lockdown. Additionally, to the best of our knowledge, no studies have considered the possible influence of gender and work type (telework vs. in person) in the proposed association, although some research has found significant differences among the covariates in the psychological distress dimensions (Naser et al., 2020; Bell et al., 2021).

The purpose of the current study is to determine whether psychological distress (i.e., depression, anxiety, and depression) is associated with coping strategies and consequently with optimism in Portuguese workers. This study will add to previous research in health psychology, as it considers the association between each psychological distress and adaptive and maladaptive coping strategies. Previous studies that have collected data with workers have rather assumed that psychological distress should operate as one unifying factor rather than dimensions of mental health.

Importantly, we will examine the relations between depression, anxiety, stress, coping, and optimism in an underresearched group but for whom optimism is likely to be of the utmost importance, that is workers, both telework and in person, who faced challenges due to the pandemic. Indeed, these individuals, despite facing daily challenges related to their working conditions, have sustained the high and long-term engagement necessary to maintain working capacity, suggesting that high levels of adaptive coping may be at play for many of these individuals and their view toward a better quality of life in the future.

We hypothesized that worker perceptions of adaptive coping would be positively associated with optimism but not maladaptive coping. Moreover, we hypothesized that depression, anxiety, and stress would be positively associated with maladaptive coping, but not with adaptive coping and optimism.

## Materials and Methods

### Participants

The inclusion criteria to participate in this study were: (a) being at least 18 years old; and, (b) agreeing to participate voluntarily in this study. Data were collected after the second lockdown, between March and June of 2021. A sample of 274 Portuguese workers (female = 54) with a mean age of 40.86 (SD = 0.70) years and mean work experience of 19.68 (SD = 12.07) years met inclusion criteria and agreed to participate in the proposed study. They represented a variety of working classes (i.e., arts, engineering, marketers, transportation and logistic, clerks, personal trainers, managers, lawyers, accountants, directors, journalism, health care). Details are reported in Table 1.

**TABLE 1:** Demographic characteristics.

Variable	Categories	M (SD)/N (%)
Gender	Female	54 (19.7)
	Men	230 (80.3)
Age	Years	40.86 (0.70)
Professional Experience	Years	19.68 (12.07)
Contract	Fixed-term	104 (38.0)
	Full-time for an indefinite period	155 (56.5)
	Self-employed contract	15 (5.5)
Sector	Accountant and finance	26 (9.5)
	Management and consultant	30 (10.9)
	Charity and voluntary work	10 (3.6)
	Art and design	10 (3.6)
	Energy	7 (2.6)
	Engineering and transformation	69 (25.2)
	Health	21 (7.7)
	Technology	4 (1.5)
	Marketing	6 (2.2)
	Media and journalism	7 (2.6)
	Sports and physical activity	5 (1.8)
	Transportation and logistics	10 (3.6)
	Education	12 (4.4)
	Commerce	40 (14.6)
	Administration	16 (5.8)
Work type	Telework	114 (41.6)
	In person	160 (58.4)

*M = Mean; SD = Standard Deviation; N = sample.*

The *A priori* sample size calculator for hierarchical multiple regression analysis (Soper, 2021) was used to calculate the minimum required participants for this study. The following inputs were used: anticipated effect size = 0.03; desired statistical power = 0.95; number of predictors in block 1 = 2; number of predictors in block 2 = 3; probability level = 0.05. The results suggested a minimum of approximately 120 participants, which provided support that the current sample size is acceptable.

### Instruments

Participants completed the Brief COPE Portuguese version (Maroco et al., 2014) due to the sample characteristics. This version contains 28-items distributed on 14 factors, in which participants responded to each item using a Likert-type response scale anchored between 1 (*Never did this*) and 5 (*Always do this*). Means were calculated for each coping factor and composite scores were calculated for adaptive (i.e., positive reframing, active coping, acceptance, religion, planning and seeking social support, use of emotional and instrumental support, and humor) and maladaptive (i.e., venting, behavioral disengagement, denial, self-distraction, and self-blame) coping dimensions according to several authors (Meyer et al., 2001; Su et al., 2015; Teques et al., 2018). Results from confirmatory factor analysis supported the measurement model (Comparative Fit Index = 0.956, Tucker–Lewis Index = 0.943, Standard Mean Root Square Residual = 0.041, Root Mean Square Error of Approximation = 0.047 (90% confidence interval = [0.030, 0.054]).

Participants completed the Optimism Scale Portuguese version (Barros, 1998) which contains 4 items measuring optimism using a Likert-type scale anchored from 1 (*totally disagree*) and 5 (*totally agree*). Results from confirmatory factor analysis supported the measurement model (Comparative Fit Index = 0.987, Tucker–Lewis Index = 0.980, Standard Mean Root Square Residual = 0.031, Root Mean Square Error of Approximation = 0.033 (90% confidence interval = [0.026, 0.037]).

Participants completed the Depression Anxiety Stress Scale Portuguese version (Pais-Ribeiro et al., 2004) which comprises measures of depression (7 items), anxiety (7 items), and stress (7 items). Each item is scored from 0 (*did not apply to me at all*) to 3 (*applied to me very much or most of the time*) and mean scores were calculated for each factor. Results from confirmatory factor analysis supported the measurement model (Comparative Fit Index = 0.921, Tucker–Lewis Index = 0.911, Standard Mean Root Square Residual = 0.053, Root Mean Square Error of Approximation = 0.063 (90% confidence interval = [0.057, 0.078]).

### Procedures

Data collection was conducted following the Declaration of Helsinki and the approval of the Institutional Research Ethics Committee of the Polytechnic Institute of Leiria (reference number: CE/IPLEIRIA/17/2021) was obtained before data collection. A non-probabilistic sampling technique to collect data was used; specifically, data were collected from a convenience sample of the population. The participants had online access to the questionnaire created for the study using Google Forms, which was promoted using digital media (e.g., social networks, academic e-platforms). All participants were informed about the main objective and goals of the study, and 18 years old or older individuals provided written informed consent before completing the questionnaire.

### Statistical Analysis

Analyses were performed in IBM SPSS Statistics v.27. No missing data at the item level was found due to how the questionnaire was built. Cutoffs for normality were determined, accepting scores were within −2/ + 2 and −7/ + 7 for skewness and kurtosis, respectively. The presence of outliers was also verified.

Next, we performed a t-test for independent samples and ANOVA to test for any differences between men and women and work type on optimism, adaptive and maladaptive coping, depression, anxiety, stress, and work experience. If differences were detected, we planned to include these variables as covariates.

Finally, hierarchical multiple regression analyses were conducted to test the proposed associations, while controlling for work experience. We considered work experience as a covariable because of its documented impact on several mental health dimensions (Lenzo et al., 2021; Serrão et al., 2021). Before performing regression analysis, tolerance test and Variance Inflation Factor (VIF) scores were analyzed to test for possible collinearity issues. The tolerance of independent variables should be greater than 0.1 for there to be no multicollinearity. The tolerance values of our study ranged from 0.39 to 0.92. In addition, the VIF should be less than 10 and our value ranged from 1.09 to 2.53. Therefore, there was no multicollinearity issue in this analysis.

## Results

### Preliminary Analysis

Descriptive statistics and the correlation matrix are reported in Table 2. Results displayed no violations of the univariate distribution since skewness and kurtosis were contained between −2 and + 2, and −7 and + 7, respectively. Three outliers were found. These extreme scores were transformed into scores no further than 3 standard deviations from the sample mean as recommended. Descriptive statistics revealed that mean scores for all factors were above the scale mid-point. Depression was positively and significantly correlated with anxiety, stress, and maladaptive coping, and negatively correlated with optimism. Similarly, anxiety was positively and significantly correlated with stress and maladaptive coping. Stress was positively correlated with both types of coping strategies, but only adaptive outcomes were positively and significantly correlated with optimism. Moreover, t-test and ANOVA did not reveal any difference between genders or working types on all main variables (ps > 0.25). Gender and working types were thus not considered as covariates.

**TABLE 2:** Descriptive statistics and correlations.

Factors	M	SD	S	K	1	2	3	4	5	6
1. Depression	1.51	0.53	1.65	3.63	1					
2. Anxiety	1.47	0.50	1.76	4.46	0.71**	1				
3. Stress	1.78	0.54	0.65	0.91	0.66**	0.59*	1			
4. Adaptive Coping	3.22	0.58	−0.46	1.12	0.01	0.06	0.14*	1		
5. Maladaptive Coping	2.35	0.48	0.81	3.14	0.33**	0.33**	0.38**	0.30**	1	
6. Optimism	4.01	0.71	−0.40	−0.23	−0.13*	0.11	−0.06	0.33**	−0.01	1

M = Mean; SD = Standard Deviation; S = Skewness; K = Kurtosis; *p > 0.05; **p > 0.01.

### Hierarchical Multiple Regression

The result of the hierarchical multiple regression is presented in Table 3. First, we checked the significance (*p*-value) of the model to examine whether the model is significantly different from a null hypothesis. Second, check the *R*^2^ value to see how much of the variance is explained by the model. Third, to identify which variable contributed to the model, we checked the significance of the variable. To examine how much variance is accounted for by the variable, we checked the standardized coefficients. In hierarchical multiple regression, we compare the models as variables are added (changes in *R*^2^).

**TABLE 3:** Hierarchical regression analysis.

	Model 1			Model 2		
	B (β)	SE	*R* ^2^	B (β)	SE	*R* ^2^
Step 1			0.125			0.211
Constant	2.98	0.26				
Adaptive Coping	0.45 (0.37)*	0.07		0.43 (0.36)*	0.09	
Maladaptive Coping	−0.18 (−0.12)*	0.09		−0.16 (−0.11)*	0.10	
Step 2						
Constant	-	-		3.01	0.26	
Depression	-	-		−0.43 (−0.32)*	0.12	
Anxiety	-	-		0.57 (0.41)*	0.11	
Stress	-	-		−0.13 (−0.10)*	0.10	

*p < 0.01.

In the first model, the regression model with the exclusion of the covariates model was statistically significant and showed that adaptive and maladaptive coping strategies accounted for 12,5% of the variance in optimism, F (2,271) = 19.303, *p* < 0.001, *R*^2^ = 0.125, adjusted *R*^2^ = 0.118. The second model accounted for 21.1% of the variance in optimism, F (3,268) = 9.773, *p* < 0.001, *R*^2^ = 0.211, adjusted *R*^2^ = 0.196, after controlling for working experience based on literature review. The change of *R*^2^ between the first and second models (Δ *R*^2^ = 0.086) was statistically significant. These results indicate that the second model is parsimonious.

Adaptive coping was positively and significantly correlated with optimism but maladaptive outcomes were negatively and significantly associated with optimism in both models. Depression was negatively and significantly associated with optimism, but the anxiety was positively and significantly correlated with optimism.

## Discussion

The study aimed to examine the associations between depression, anxiety, stress coping strategies (i.e., adaptive and maladaptive cope), and optimism. Our findings indicated that adaptive coping and anxiety are positively and significantly associated with higher expectations toward the future. In contrast, maladaptive coping and depression were both negatively associated with reduced optimism.

Based on bivariate correlations, all three psychological distress dimensions were positively and significantly associated with maladaptive coping, but only stress was positively and significantly associated with adaptive coping. However, hierarchical multiple regression analysis suggests that depression and maladaptive coping may be more important for Portuguese workers to feel lower levels of optimism, while anxiety and adaptive coping may be more crucial for these individuals to feel higher positive expectations regarding the future.

### Psychological Distress and Coping

The present study identified the coping strategies associated with psychological distress among Portuguese workers, while controlling for gender, working experience and work type. T-test for independent samples and ANOVA did not found significant differences in depression, anxiety, and stress. Nonetheless, these psychological stress dimensions were positively and significantly associated with maladaptive coping. In consistency with our results, previous studies have shown that individuals with mood disorders used significantly more maladaptive coping strategies in workers during COVID-19 lockdown (Mishra et al., 2021; Salazar et al., 2021) and after (Almeida et al., 2021). Our results and these studies support our hypothesis that, as individuals experience feelings of self-doubt, severe despondency, and sadness, tend to void situations, events, damaging self-development, restricting growth, and failing to experience well-being.

Contrarily to what was hypothesized, that depression, anxiety, and stress would be negatively and significantly correlated with adaptive coping, anxiety was positively and significantly correlated with adaptive coping. Salazar et al. (2021) also found a positive association between anxiety and instrumental support (form of adaptive coping), suggesting that anxiety may not always be considered as an negative determinant of coping. Individuals who have dealt with stressful events such as COVID-19 lockdown and associated consequences may be more empathetic and understanding to the issues that other people may have faced (Salazar et al., 2021). Thus, there could be some positive thinking that helps individuals deal with anxiety in a more adaptive manner, as the focus shifts from negative thoughts or concerns to active coping and searching for support (Almeida et al., 2021). It is worth to mention that anxiety levels are nonetheless low (i.e., below midpoint) and that the correlation with adaptive coping is weak.

### Coping and Optimism

Current findings suggests that maladaptive coping displayed a negative association whith optimism, whereas adaptive coping displayed a positive association whith optimism. This result aligns with our hypothesis and previous research (Leslie-Miller et al., 2021; Smida et al., 2021). Individuals who were motivated to deal with COVID-19 displayed higher so cews in optimism and positive anticipation (Leslie-Miller et al., 2021). A clear set of adaptive coping skills, including how to think optimistically and how to approach problems and adverse events, has been shown to help workers deal with psychological distress (Smida et al., 2021). Hence, individuals making reasonably realistic appraisals of problems and planning the future, and trying to prevent adverse effects of existing events may provide guidance to a more positive view of the future. Portuguese workers who put effort to manage stress, use active cope, recognizes the adverse event may be more prone to adapt to the current situation and search for a better quality of life in the future.

### Strengths, Limitations, and Agenda for Future Research

To the best of our knowledge, this study was the first to examine coping strategies, psychological distress, and optimism among Portuguese workers during the COVID-19 pandemic. Our study involved several labor activities which present a heterogeneous sample. Portuguese validated measures were used which presents a strength in this research. Nevertheless, we acknowledge some limitations. First, the present study was conducted with a relatively small sample. Although the sample was large enough to achieve adequate statistical power and collecting data from targeted samples such as workers presents additional difficulties and barriers, a larger sample size in future studies may have yielded greater external validity. Second, the cross-sectional design does not allow to examine changes over time or to draw causality. While to date there are virtually no studies that have conducted experimental research protocols, there is a need to apply interventions that endorse adaptive outcomes as a means to decrease psychological distress and increase optimism. Third, this research was limited to Portuguese workers, and findings cannot be easily generalized to other countries. Last, being a self-reported study, the possibility of reporting bias, especially when the pandemic could have led to different subjective experiences. Fourth, considering sample size and sampling method, composite scores for adaptive and maladaptive scores were considered in the regression analysis and discussion. While coping strategies were computed as composite scores in the model based on previous evidence (Meyer et al., 2001; Teques et al., 2018), future studies should collect data from a larger sample as a mean to provide sufficient statistical power to examine the independent contribution of each coping strategy on optimism.

### Practical Implications

The present research has several implications for health psychologists involved in public health dealing with essential workers and those working from home. First, our findings suggest that adaptive coping is a significant contributor to faith and expectations toward the future. Means on adaptive coping and optimism were indeed high, suggesting that most workers could be adopting adaptive coping strategies in their lives having a significant contribution in their workplace. Second, it seems that anxiety may not always be an indication of psychological distress, but rather a strong desire or concern to do something or for something to happen. A psychologist by measuring psychological distress could examine in further detail how anxiety is affecting their work and overall well-being and thus provide effective and timely inputs that assist workers with adopting coping mechanisms that emphasize accomplishments and provide satisfaction with life (Almeida et al., 2021).

While this study provided significant insights considering the applied relevance of the findings in the health psychology domain, this was not an intervention study. Thus, we do not know whether inducing a particular coping response will have the same effect in all working activities and countries. Nonetheless, current results showed that positive reframing, active coping, acceptance, religion, planning and seeking social support, use of emotional and instrumental support, and humor may have a significant contribution for promoting optimism. That is, search for support, planning and seeking strategies for greater well-being and accepting current situation provides positive judgment regarding life and feeling good some sort of comfort.

## Conclusion

The findings of our study may be a starting point for the exploration of the linkage between perceived psychological distress, coping strategies, and optimism when people are facing challenges after COVID-19 lockdown. People who dealt with stress and anxiety in a relatively difficult environment may tend to use more adaptive coping mechanisms, which in turn can lead to increased levels of perceived optimism related to the future.

In sum, the present study provides additional support for the significant role of adaptive coping strategies for promoting optimism as well as demonstrates the unique importance of anxiety for Portuguese workers. Anxiety may in some instances signal that something is very important for the individual and that good things will happen and that their desires or aims will ultimately be fulfilled. Workers persist when depressive symptoms and stress are low, and when they adopt adaptive coping strategies in a way that promotes well-being. Further research is, however needed to increase knowledge on the processes linking coaching behaviors, basic needs, and life satisfaction in the context of work.

## Data Availability Statement

The datasets analyzed during the current study are not publicly available due to the funding project but are available from the corresponding author upon request.

## Ethics Statement

The studies involving human participants were reviewed and approved by CE/IPLEIRIA/17/2021. The patients/participants provided their written informed consent to participate in this study.

## Author Contributions

TS and FR conceived this manuscript, led the writing team, conducted the study search, summarized the quantitative review, and drafted the “Results” section. AU, JR, and A-HM made substantial contributions to the “Discussion” section. RF, DM, and FR revised the entire manuscript and made important contributions in various sections. All authors read and approved the final version of the manuscript.

## Conflict of Interest

The authors declare that the research was conducted in the absence of any commercial or financial relationships that could be construed as a potential conflict of interest.

## Publisher’s Note

All claims expressed in this article are solely those of the authors and do not necessarily represent those of their affiliated organizations, or those of the publisher, the editors and the reviewers. Any product that may be evaluated in this article, or claim that may be made by its manufacturer, is not guaranteed or endorsed by the publisher.
